# Supplementary figures and images for: Characterisation of two Toxoplasma PROPPINs homologous to Atg18/WIPI suggests they have evolved distinct specialised functions
Source: PLoS One. 2018 Apr 16;13(4):e0195921. doi: 10.1371/journal.pone.0195921 (PMC5901921; doi:10.1371/journal.pone.0195921)

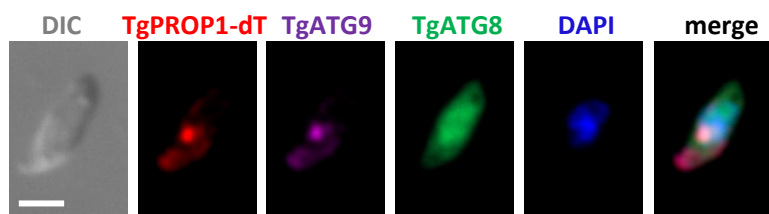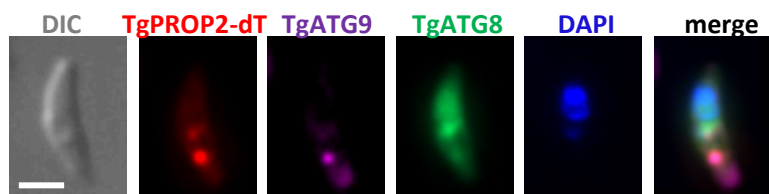

Supplement: S1 Fig — A cell line stably expressing HA-tagged TgATG9 [24] was co-transfected with plasmids for expressing either TgPROP1-dT or TgPROP2-dT together with GFP-TgATG8. 24 hours post-transfection parasites were mechanically released from their host cells and incubated in amino acid-depleted medium for 6 hours before being fixed, adhered to poly-L-lysine slides, and processed for immunofluorescence. Scale bar = 2 μm. (PDF) [file pone.0195921.s001.pdf]

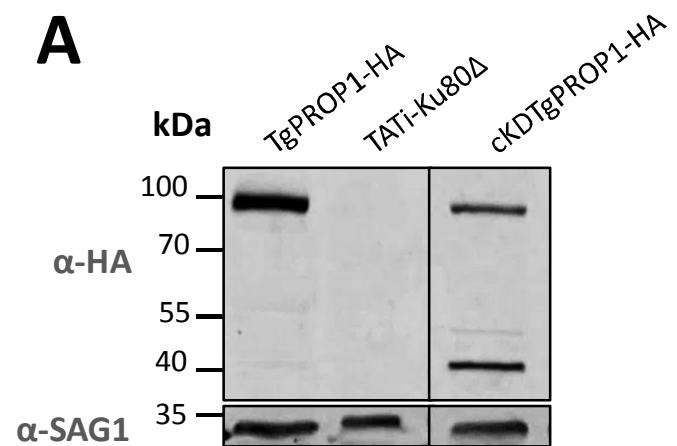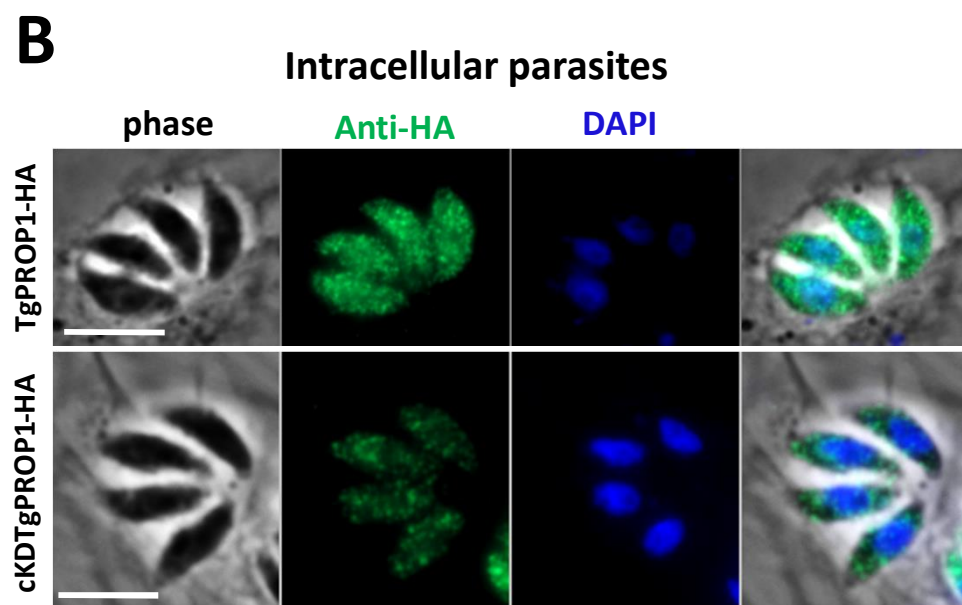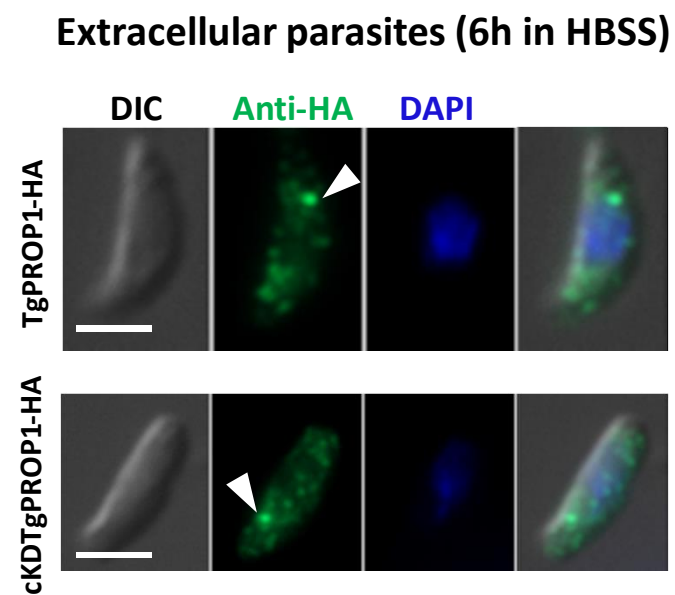

Supplement: S2 Fig — A) Immunoblot analysis of the HA-tagged TgPROP1 expressed with its own promoter (TgPROP1-HA) or after replacement with a SAG4 promoter (cKD-TgPROP1-HA). SAG1 was used as a loading control. B) Localisation of HA-tagged TgPROP1 expressed with its own promoter (TgPROP1-HA) or after replacement with a SAG4 promoter (cKD-TgPROP1-HA) in intracellular (left) or starved extracellular (right) parasites. DNA was stained with DAPI. Scale bar = 5 μm (left) or 2 μm (right). (PDF) [file pone.0195921.s002.pdf]

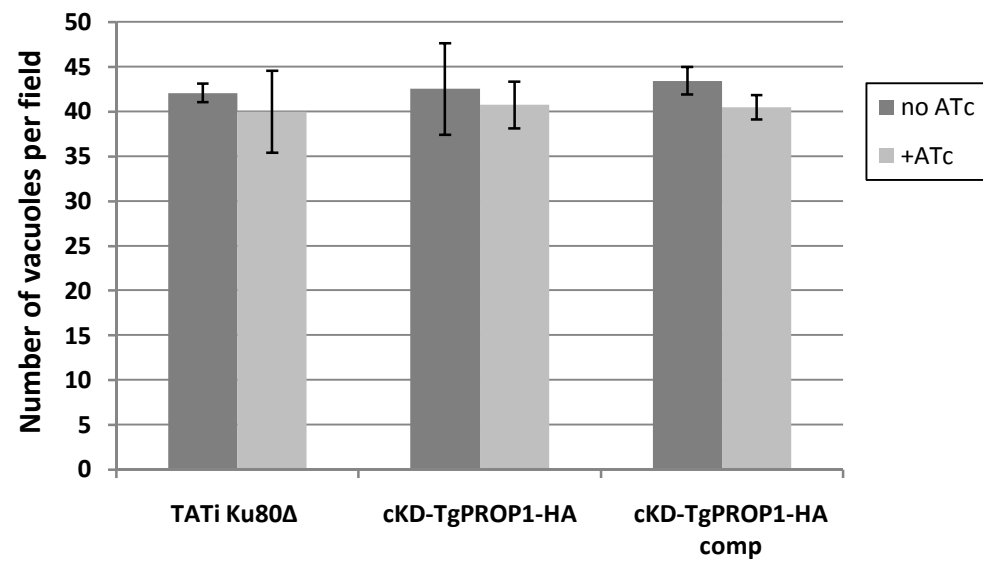

Supplement: S3 Fig — TATi-Ku80Δ, cKD-TgPROP1-HA and cKD-TgPROP2-HA parasites were mechanically released from their host cells and assessed for their ability to invade host cells. (PDF) [file pone.0195921.s003.pdf]

**A**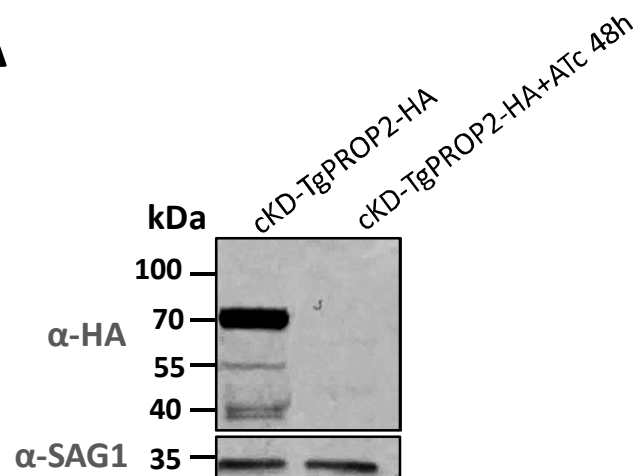**B**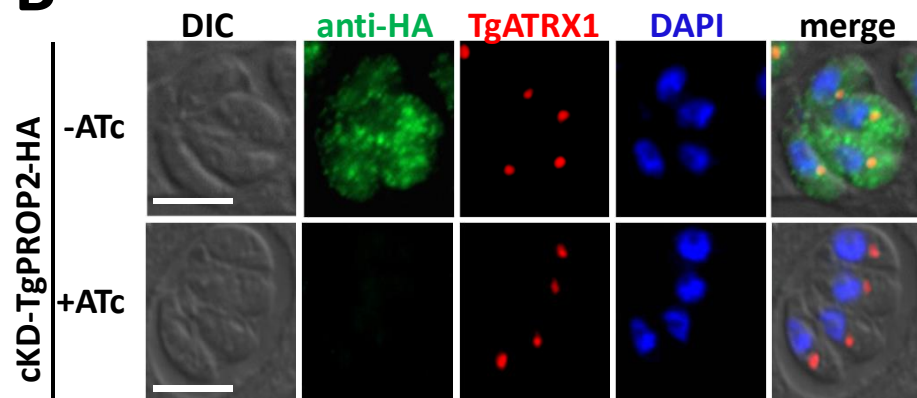**C**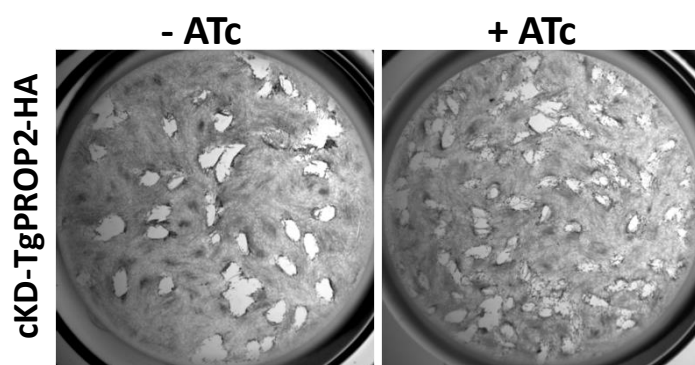**D**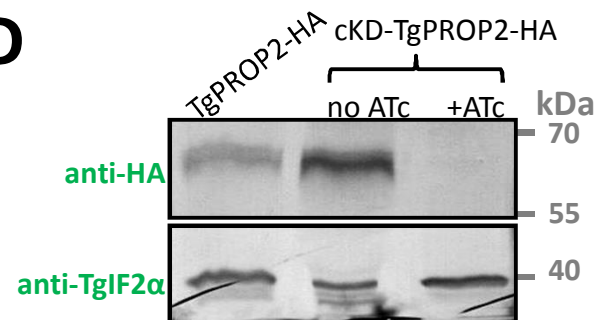**E**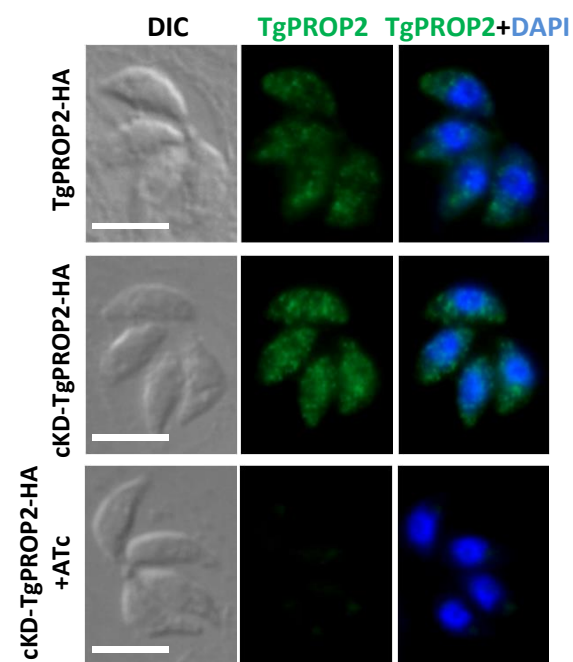**F**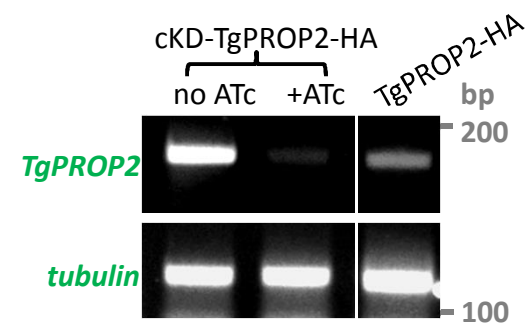

Supplement: S4 Fig — A) Immunoblot analysis of TgPROP2-HA depletion after two days of ATc incubation. SAG1 was used as a loading control. B) IFA of TgPROP2-HA depletion after two days of ATc incubation. TgPROP2 was detected with anti-HA antibodies, the apicoplast was detected using anti-TgATRX1 antibodies. DNA was stained with DAPI. Scale bar = 5 μm. C) Plaque assay show conditional depletion of TgPROP2 has no drastic effect on the lytic cycle. D) Immunoblot analysis shows promoter change leads to an overexpression of TgPROP2-HA. TgIF2α was used as a loading control. E) IFA also shows a higher level of TgPROP2-HA expression when expressed from the SAG4 promoter. DNA was stained with DAPI. Scale bar = 5 μm. F) Semiquantitative RT-PCR analysis of TgPROP2 expression shows minute amounts of mRNA are still detectable after 3 days of incubation with ATc. Analysis was performed on parasites incubated or not with ATc for 3 days regulate mRNA expression. Specific β-tubulin primers were used as controls. (PDF) [file pone.0195921.s004.pdf]

**A**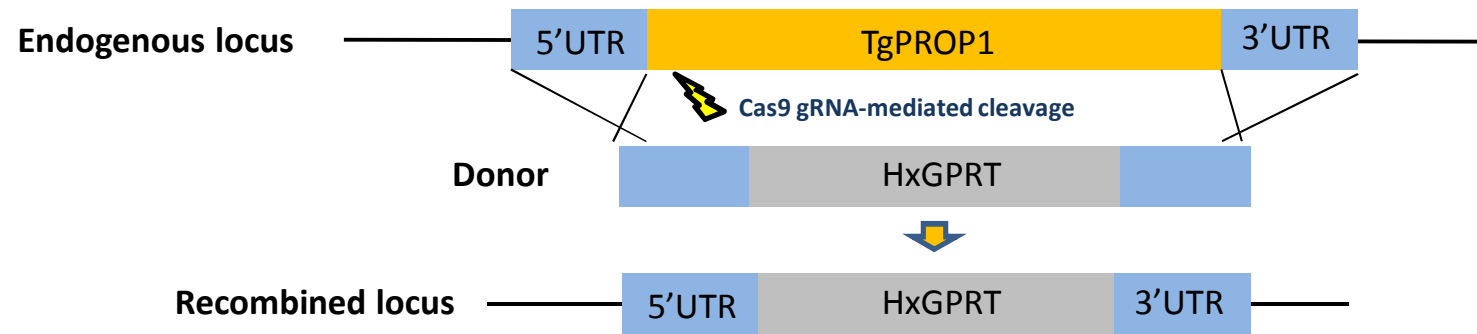**B**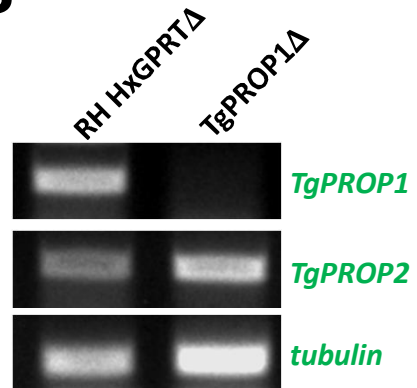

Supplement: S5 Fig — A) Schematic representation of the strategy for generating a TgPROP1 knock-out cell line using CRISPR/Cas9. Locus modification was made in the RH cell line deleted for the Hypoxanthine-guanine phosphoribosyltransferase (HxGPRT) gene. The donor sequence contained a HxGPRT sequence for selection of transgenic parasites with mycophenolic acid and xanthine. B) RT-PCR analysis showing efficient TgPROP1 mRNA depletion in the TgPROP1Δ cell line. Specific TgPROP2 and β-tubulin primers were used as controls. (PDF) [file pone.0195921.s005.pdf]

**A**Cas9 – *TgPROP2* guide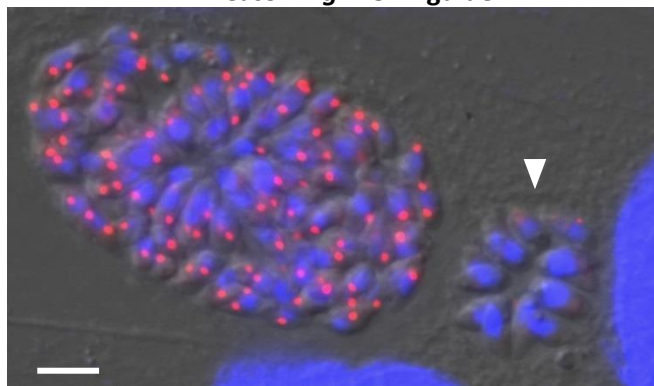

Cas9 – no guide

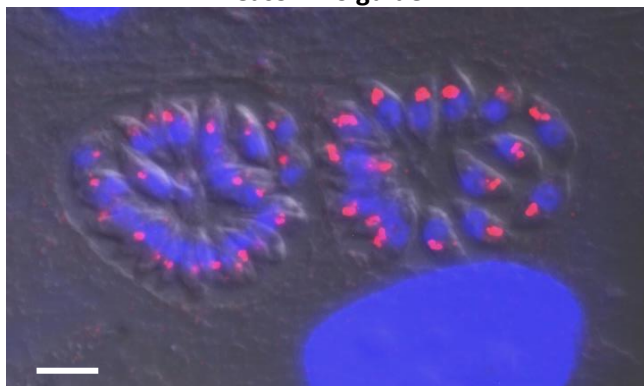**B**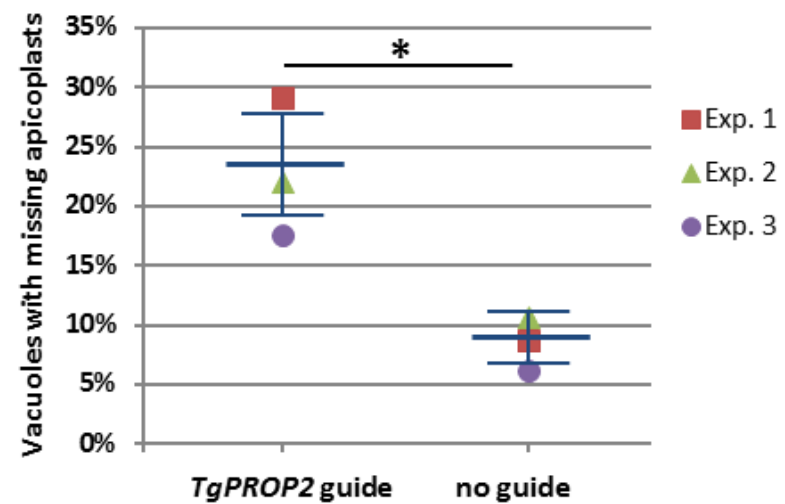

Supplement: S6 Fig — A) IFA shows apicoplast loss in a vacuole (arrowhead) 48 hours after co-transfection of a vector expressing Cas9 and a guide RNA specific of TgPROP2 together with a donor sequence for TgPROP2 inactivation (left), or a the donor sequence and the Cas9-expressing vector without the guide sequence as a control (right). Scale bar = 5 μm. B) Quantification of apicoplast loss in vacuoles 48 hours after transfection of tachyzoites of the RH strain, in conditions described in A). 100 vacuoles were counted. Values from three independent experiments, as well as mean ± SEM, are represented. The asterisk denotes a significant statistical difference as confirmed by Student’s t-test (p<0.05). (PDF) [file pone.0195921.s006.pdf]
